# Migalastat Versus Enzyme Replacement Therapy in Treatment‐Naïve Fabry Disease: Real‐World Evidence From the followME Fabry Pathfinders Registry

**DOI:** 10.1002/jimd.70261

**Published:** 2026-11-09

**Authors:** Peter Nordbeck, Agnieszka Kopiec, Noemi Hummel, Vera Gielen, Biliana O. Veleva‐Rotse, Alasdair MacCulloch

**Affiliations:** ^1^ University Hospital Würzburg Würzburg Germany; ^2^ Certara Krakow Poland; ^3^ Certara GmbH Lörrach Germany; ^4^ BioMarin UK Ltd London UK; ^5^ BioMarin Pharmaceutical Inc. Novato California USA

**Keywords:** enzyme replacement therapy, estimated glomerular filtration rate, Fabry disease, Fabry‐associated clinical event, migalastat, real‐world effectiveness

## Abstract

Fabry disease is a rare, progressive, X‐linked disease resulting from α‐galactosidase A enzyme deficiency due to *GLA* gene variants. Current treatments include intravenous enzyme replacement therapy (ERT) and the oral pharmacologic chaperone migalastat. The followME Fabry Pathfinders registry (EUPAS20599) is an ongoing prospective patient registry evaluating outcomes for Fabry treatments. The present analyses compared the effectiveness of migalastat with ERT in treatment‐naïve patients ≥ 16 years at enrollment. The prespecified primary outcome was Fabry‐associated clinical event (FACE) rates/1000 person‐years from treatment start. Secondary outcomes included estimated glomerular filtration rate (eGFR) annualized rate of change, proportion of patients with FACEs, FACE incidence rates, and time to first FACE. The populations included 319 migalastat‐ and 140 ERT‐treated patients for FACE analyses; 268 and 119, respectively, for eGFR_CKD‐EPI_ outcomes. Propensity score matching (1:1, nearest neighbor with replacement) balanced treatment groups with absolute standardized mean differences < 0.1 after matching for baseline age, sex and eGFR (matching for amenability status was not possible). Overall, patients experienced 55.3 FACEs/1000 patient‐years (95% confidence interval [CI] 40.7, 75.3) with migalastat and 100.7 (53.7, 188.9) with ERT; the event‐rate ratio was 0.55 (0.27, 1.11; *p* = 0.093). The event‐rate ratio (95% CI) for cerebrovascular FACEs was 0.24 (0.06, 0.88; unadjusted ***p* = 0.031**; Benjamini‐Hochberg‐adjusted *p* = 0.093), representing 4.7 (2.0, 11.2) FACEs/1000 patient‐years with migalastat and 19.8 (7.5, 52.4) with ERT. eGFR_CKD‐EPI_ slope, mL/min/1.73 m^2^/year (95% CI), was −1.47 (−2.17, −0.77) with migalastat and −2.50 (−3.53, −1.46) with ERT. Comparisons generally numerically favored migalastat over ERT, with cerebrovascular FACEs being statistically significant when not adjusting for multiple comparisons. These analyses support real‐world multisystemic effectiveness of migalastat in Fabry disease.

## Graphical abstract

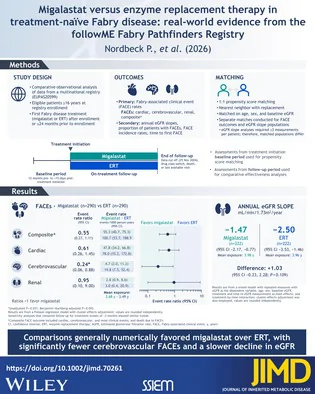

## 1 Introduction

Fabry disease is a rare, progressive, X‐linked inborn error of metabolism that leads to α‐galactosidase A (α‐gal‐A) enzyme deficiency or absence due to *GLA* gene variants [1]. This deficiency causes cellular dysfunction, a progressive accumulation of the neutral glycosphingolipid, globotriaosylceramide (Gb3), inflammation, cell death, and eventual multisystem organ damage and failure. Fabry‐associated clinical events (FACEs), particularly in the renal, cardiac, and cerebrovascular systems, result in significant morbidity and mortality, and a key treatment goal is preventing these events [2]. Classic Fabry disease occurs when there is minimal or no enzyme function and manifests in childhood or adolescence. Most patients have later‐onset Fabry disease, which has a more heterogeneous progression pattern compared to classic [3]. Despite its X‐linked nature, both males and females usually develop clinically significant disease manifestations over time [4, 5].

Enzyme‐replacement therapy (ERT) is a bi‐weekly infusion of recombinant α‐gal‐A enzyme that promotes the degradation of Gb3 in lieu of the defective endogenous enzyme and can delay disease progression and improve long‐term outcomes [6, 7]. ERT therapy is limited by the burden of infusions every 2 to 4 weeks, the potential for immunogenicity, and the low blood–brain barrier penetration [8]. Approved enzyme‐replacement therapies (ERT) for Fabry disease include agalsidase alfa approved in the European Union (EU) and other countries but not in the US; and agalsidase beta and pegunigalsidase alfa approved in the US, EU, and other countries.

The small molecule pharmacological chaperone migalastat is an effective oral treatment for Fabry disease which works with the endogenously produced enzyme. Migalastat binds reversibly to the active site in amenable α‐gal‐A variants and restores folding and stability which results in increased enzyme activity due to improved trafficking to the lysosome and decreased degradation of the mutated enzyme [8, 9, 10]. Migalastat is approved in the US, EU and other countries for patients with Fabry disease who have amenable *GLA* gene variants. In the EU, migalastat has been approved for use for 10 years. In 2 pivotal randomized, multi‐center, controlled Phase 3 clinical trials and in real‐world effectiveness studies, migalastat was shown to stabilize renal function and cardiac size, and was associated with low FACE rates in patients with Fabry disease [11, 12, 13, 14, 15, 16, 17, 18]. However, there are limited studies investigating migalastat vs. ERT in Fabry disease, thus the comparative effectiveness is ultimately unclear.

The followME Fabry Pathfinders registry (EUPAS20599) is an ongoing, prospective, observational, multicenter, multinational registry enrolling untreated, ERT‐treated, and migalastat‐treated patients with Fabry disease [19]. The registry was established in 2018 and collects data on safety and effectiveness including renal function, FACEs, serious adverse events, and overall survival. Primary registry outcomes are safety and FACEs.

To date, the real‐world effectiveness of migalastat compared to ERT has not been evaluated in matched populations. In this study, which aimed to address this evidence gap, data were extracted from the followME Fabry registry to compare rates of FACEs and the annualized rate of change in eGFR in matched patients treated with migalastat versus ERT.

## 2 Materials and Methods

### 2.1 Study Design and Data Source

Data for patients whose first treatment was migalastat or ERT were extracted from the followME Fabry registry with a data cut off of November 25, 2024. The index date was defined as the start date of either migalastat or ERT. Baseline was defined as the period from 12 months prior to the index date until 15 days post index date. If there was > 1 assessment with available data during the baseline period, the assessment closest to the index date was selected. In case of two equidistant values, the one prior to the index date was selected.

For the main analysis, for patients who did not switch to another treatment, exposure time was calculated as the difference between the patient study completion date or date of last visit if study completion date was missing, and the start date of treatment. For patients who switched to another drug class (a switch between ERTs was considered as continued ERT treatment), exposure time was calculated as the difference between the start date of the next treatment minus the start date of the first treatment minus 1 day. For the main analysis, treatment breaks were not considered.

For the sensitivity analysis, if patients did not switch to another treatment or stop the first treatment for ≤ 90 days, exposure time was calculated as the difference between the patient study completion date or date of the last visit and the start date of the first treatment. If patients switched to another drug class, exposure time was calculated as the difference between the start date of the next treatment minus the start date of the first treatment minus 1 day. If patients stopped treatment completely or for > 90 days, exposure time was calculated as the difference between the stop date of the treatment and the start date of the treatment plus 91 days.

### 2.2 Study Population

Patients from the followME Fabry registry were included in the analysis if they satisfied the inclusion criteria published previously [18], if they were ≥ 16 years of age and initiated their first treatment in the 24 months prior to enrollment or any time after enrollment without prior treatment. Migalastat‐treated patients were required to have amenable *GLA* gene variants. For the eGFR slope analysis, patients were required to have ≥ 3 eGFR assessments, including the baseline assessment, to be included in the analysis.

Exclusion criteria included any patients who had participated in a clinical trial of any investigational product or device at the time of enrollment or had failed to meet eligibility requirements, had a protocol violation, or withdrew at enrollment.

The complete list of inclusion and exclusion criteria is provided in Table S1.

### 2.3 Study Outcomes

The primary outcome was FACE rates per 1000 person‐years from the index date. FACEs were defined as cardiac (myocardial infarction, new symptomatic arrhythmias requiring medication, unstable angina, congestive heart failure requiring hospitalization, or any major cardiac medical procedure), cerebrovascular (stroke or transient ischemic attack), and renal (sustained doubling of serum creatinine from baseline on two consecutive visits, end‐stage kidney disease requiring long‐term dialysis or transplantation) clinical events and death due to FACEs (recorded based on the FACE category). Secondary outcomes included annualized rate of change in eGFR where eGFR was calculated using the Chronic Kidney Disease Epidemiology Collaboration equation (eGFR_CKD‐EPI_) [20], proportion of patients with FACEs, FACE incidence rates, and time to first FACE.

FACE rate reported as number of events per 1000 person‐years was calculated as follows:

$$\frac{n_{\text{FACE}}}{\sum_{i}T_{i}}$$

where *n*_FACE_ is the total number of FACE(s) and *T*_i_ is the total exposure time of patient *i* so that all FACEs falling within the treatment period were counted, and patients were followed from index date until study end date, change/switch in therapy, death or loss to follow‐up, whatever came first.

FACE incidence rate reported as number of patients per 1000 person‐years was calculated as follows:

$$\frac{N_{\text{FACE}}}{\sum_{i}t_{i}}$$

where *N*_FACE_ is the number of patients with FACE(s) and *t*_*i*_ is the exposure time of patient *i* from index date until date of first FACE event, study end date, change/switch in therapy, death, or loss to follow‐up, whatever came first. i.e., if a patient has multiple FACEs, *t*_*i*_ is the time of the first FACE from the treatment start date, so that for each patient with FACE(s), only the first FACE was counted.

The proportion of patients with FACEs was defined as the ratio of patients with FACEs on a given therapy divided by the number of patients on that therapy. Time to first FACE was defined as the time from index to the first FACE in years. Patients who completed the study, were lost to follow‐up, or discontinued the study without an event were censored at the patient study completion date (date of last visit if study completion date was missing); patients who switched therapy without an event prior to the switch were censored at the day preceding the start date of the next treatment.

eGFR was derived from serum creatinine values and age at index using the CKD‐EPI 2009 equation [20].

### 2.4 Statistical Analysis

Sample size was determined using composite clinical outcome rates per 1000 patient‐years from the ATTRACT trial: 428 and 791 composite clinical outcomes per 1000 patient years for migalastat and ERT, respectively [13]. It was assumed that the event rates in followME would be 50%–75% lower due to the use of an updated definition of FACEs vs. ATTRACT, which is more appropriate for long term clinical monitoring compared with the prior definition [21]. Therefore, a sample size of 87–174 migalastat and 44–87 ERT patients was deemed to be sufficient to observe an effect between the groups with 99% power at a 5% 2‐sided significance level, based on the Poisson distribution [22]. A prior feasibility assessment found that the sample size would be sufficient based on enrollment to determine a difference in migalastat vs. ERT in the primary outcome.

In case serum creatinine at index (used to derive eGFR) was missing, the baseline period was broadened to 1.5 years before and 100 days after the index date to reduce the percentage of missingness. Remaining missing serum creatinine values were imputed using the fully conditional specification method, including age at index and sex, BMI, urine creatinine, and hypertension as variables in the regression model. Missing dates were imputed as follows: in case only year and month were reported, the day was set to the 15th of the month; if only year was given, then the month and day were set to 1st July.

Propensity score (PS) matching was used to account for differences between migalastat‐ and ERT‐treated patient groups. Ideal variables considered for inclusion in the propensity score matching were age at index, eGFR at index, sex, left ventricular mass index, urine protein to creatine ratio, angiotensin II receptor blocker or angiotensin converting enzyme inhibitor use at index, history of FACEs, and Fabry phenotype [23, 24], if < 25% of the data for the respective variable was missing. Fabry phenotype was not reported in the followME Fabry registry and could not be included. Angiotensin II receptor blocker or angiotensin converting enzyme inhibitor use at index and history of FACEs were not required to be reported, so it was not possible to determine their level of missingness. Therefore, these variables were not included in the propensity score matching. Each patient treated with migalastat was matched to a patient treated with ERT by 1:1 matching using a nearest‐neighbor algorithm with replacement if the difference in the logit of the propensity score was ≤ 0.1 times the standard deviation of the logit of the propensity score. This means that migalastat patients are excluded during matching when no ERT patient exists within the caliper (0.1 standard deviation of the logit of the propensity score), and conversely, ERT patients whose propensity score falls outside the range of all migalastat patients' calipers are never selected as a match and are therefore also excluded during the matching. Standardized mean differences (SMD) were calculated to examine the quality of the matching. SMDs with an absolute value of ≤ 0.1 were considered balanced. Propensity score distributions were also examined graphically with strong overlap between the 2 groups indicating good matching.

Poisson regression analysis was used to compare the event rate and incidence rate of FACEs per 1000 person‐years according to propensity score matched treatment groups. In case of over‐dispersed data, negative binomial regression was also evaluated. Event rate and incidence rate ratios were derived. Because multiple FACE outcomes were examined, unadjusted *p*‐values and *p*‐values adjusted using the Benjamini‐Hochberg (BH) false discovery rate procedure across the three organ‐specific FACE outcomes were reported [25, 26]. A Cox proportional hazards model was applied to the propensity score matched cohort to assess the time to the first FACE among all participants. Both an unadjusted model and one including the variables used for propensity score matching as covariates were applied. The estimate of the hazard ratio (HR) and 95% confidence interval (CI) for migalastat vs. ERT‐treated patients was calculated. For the proportion of patients with FACEs, risk ratios (95% CI) were provided using Poisson regression analysis.

A mixed model with repeated measures (MMRM), using the eGFR measurements directly from the propensity score matched cohort, was used to compare eGFR slope. The MMRM used eGFR as the dependent variable; treatment, time to eGFR measurement, and the variables used for propensity score matching as main effects; and treatment by time interaction. Time to eGFR measurement was defined as days from the index date to the date of eGFR measurement. A linear random coefficient model with the intercept and time to eGFR measurement was used for mean response over time. The intercept and slope were allowed to vary across study participants. Treatment differences were assessed by testing for treatment by time interaction. Since the slope estimate from the MMRM model is the rate of change in a day, the annualized rate of change in eGFR was calculated as the slope estimate times 365.25. An unstructured covariance matrix was used.

In the event that patients from the ERT cohort were matched to > 1 patient in the migalastat cohort, methods to obtain robust standard errors were used to account for the non‐independence of data grouped in clusters for the FACEs and the eGFR analyses where matching with replacement was used.

## 3 Results

### 3.1 Study Population

A total of 909 patients were enrolled in the followME Fabry registry (Figure 1). Patients were sequentially excluded from this study if they did not meet the registry's eligibility requirements (*n* = 131), were aged < 16 at enrollment (*n* = 4), participated in a clinical trial of an investigational medicinal product or device (*n* = 3), did not start migalastat or ERT within 24 months prior to or any time after enrollment (*n* = 304), were not treatment‐naïve (*n* = 6), stopped treatment prior to enrollment (*n* = 1), or were non‐amenable and treated with migalastat (*n* = 1). A total of 459 patients were eligible for this analysis. The final study population for the FACE outcomes included 459 patients, 319 (69.5%) treated with migalastat and 140 (30.5%) treated with ERT. The final study population for eGFR slope included 387 patients, 268 (69.3%) treated with migalastat and 119 (30.7%) treated with ERT. A total of 72 patients who did not have ≥ 3 eGFR assessments were excluded from the eGFR slope population.

**FIGURE 1:**
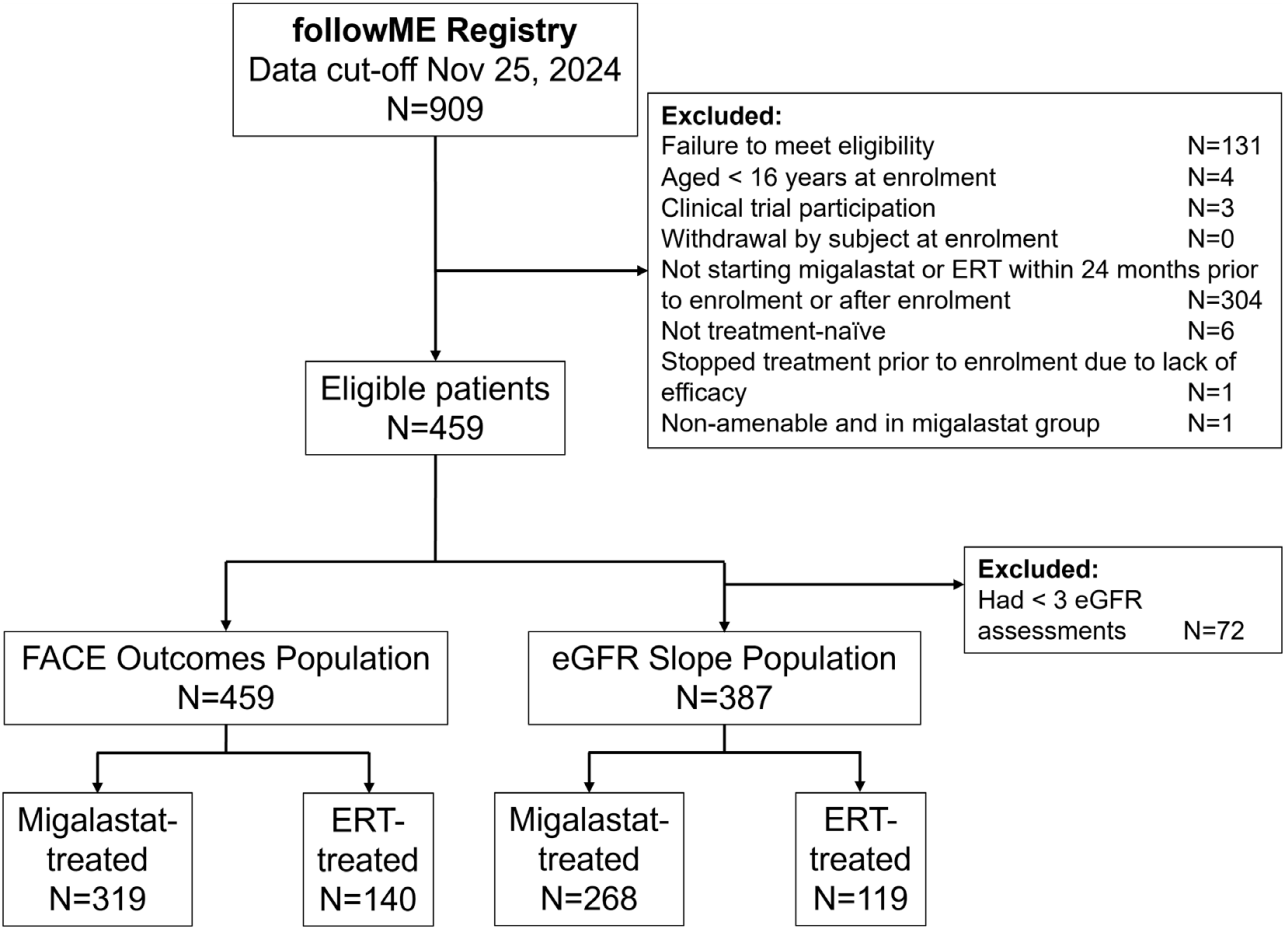
Patient flow chart. eGFR, estimated glomerular filtration rate; ERT, enzyme replacement therapy; FACE, Fabry‐associated clinical events.

For the study population for FACE outcomes, 290 (91%) migalastat‐treated patients were matched to 106 (76%) ERT‐treated patients. Most patients (approximately 75% of the included patients) had a weight of 1, 2, or 3 and the maximum weight was 20 (1 patient) for the FACE outcomes population, and 8 (1 patient) for the eGFR slope population (Table S2). Population characteristics for the FACE outcomes population are shown before and after matching in Table 1. Additional baseline characteristics including body weight, body mass index (BMI), and Fabry symptoms for the FACE outcome population are shown in Table S3. For the study population for eGFR slope, of the 268 migalastat‐treated patients, 222 (83%) were matched to 80 (67%) of the ERT‐treated patients. Population characteristics for the eGFR slope population are shown before and after matching in Table 2. Additional baseline characteristics including body weight, BMI, and Fabry symptoms for the eGFR slope outcome population are shown in Table S4.

**TABLE 1:** Study population for FACE outcomes.

Characteristics	Before matching		After matching	
	Migalastat	ERT	Migalastat	ERT
*N*	319	140	290	290
Age at index (years)				
Mean (SD)	51.3 (14.3)	46.0 (12.5)	50.9 (13.3)	50.3 (12.4)
Median (min, max)	54.0 (16.0, 78.0)	46.0 (18.0, 73.0)	53.0 (16.0, 78.0)	51.0 (18.0, 73.0)
Male sex, *n* (%)	167 (52.35%)	59 (42.14%)	150 (51.72%)	150 (51.72%)
Race, *n* (%)				
Asian	0 (0.00%)	1 (0.71%)	0 (0.00%)	0 (0.00%)
Black or African American	1 (0.31%)	1 (0.71%)	1 (0.34%)	0 (0.00%)
White	307 (96.24%)	123 (87.86%)	279 (96.21%)	244 (84.14%)
Not reported	11 (3.45%)	15 (10.71%)	10 (3.45%)	46 (15.86%)
ERT use, *n* (%)				
Agalsidase alfa	NA	56 (40.00%)	NA	96 (33.10%)
Agalsidase beta	NA	84 (60.00%)	NA	194 (66.90%)
Time since Fabry disease diagnosis^a^, years				
Mean (SD)	3.6 (5.2)	6.0 (9.2)	3.6 (5.2)	5.9 (9.3)
Median (min, max)	1.4 (0.0–35.3)	1.1 (0.0–49.0)	1.4 (0.0–35.3)	1.1 (0.0–49.0)
N missing	5	0	3	0
Age at onset of Fabry disease symptoms^b^, years				
Mean (SD)	41.6 (19.0)	26.7 (17.7)	40.9 (18.7)	28.8 (17.5)
Median (min, max)	46.5 (5.0, 74.0)	22.0 (2.0, 61.0)	45.0 (5.0, 72.0)	30.0 (3.0, 61.0)
N missing	127	22	111	81
Amenability, *n* (%)	319 (100.00%)	36 (25.71%)	290 (100.00%)	116 (40.00%)
Variant type^c^				
Frameshift variant	0 (0.00%)	17 (12.14%)	0 (0.00%)	33 (11.38%)
In‐frame deletion	2 (0.63%)	3 (2.14%)	2 (0.69%)	0 (0.00%)
In‐frame duplication	2 (0.63%)	0 (0.00%)	1 (0.34%)	0 (0.00%)
Initiation codon variant (start‐loss)	0 (0.00%)	2 (1.43%)	0 (0.00%)	2 (0.69%)
Large deletion (multi‐exon)	0 (0.00%)	3 (2.14%)	0 (0.00%)	7 (2.41%)
Missense variant	313 (98.12%)	95 (67.86%)	285 (98.28%)	214 (73.79%)
Nonsense variant (stop‐gain)	0 (0.00%)	14 (10.00%)	0 (0.00%)	20 (6.90%)
Splice site variant (canonical ±1/±2 or intronic)	0 (0.00%)	5 (3.57%)	0 (0.00%)	7 (2.41%)
Unclassified	2 (0.63%)	1 (0.71%)	2 (0.69%)	7 (2.41%)
eGFR, mL/min/1.73m^2^				
Mean (SD)	91.4 (24.0)	94.2 (21.1)	89.9 (20.7)	91.9 (18.9)
N missing	70	31	0^d^	0^d^
LVMI, g/m^2^				
Mean (SD)	109.9 (45.1)	119.9 (88.2)	108.7 (43.5)	157.4 (125.2)
Median (min, max)	98.4 (40.0–272.7)	97.6 (48.9–625.9)	96.8 (40.0–272.7)	108.3 (48.9–625.9)
N missing	201	91	183	208
Exposure time, years				
Mean (SD)	3.66 (1.69)	3.68 (1.69)	3.68 (1.70)	3.49 (1.54)
Median (min, max)	3.70 (0.10–6.99)	3.55 (0.53–6.97)	3.72 (0.10–6.99)	3.26 (0.53–6.97)

Abbreviations: eGFR, estimated glomerular filtration rate; ERT, enzyme replacement therapy; LVMI, left ventricular mass index; max, maximum; min, minimum; NA, not applicable; SD, standard deviation.

^a^ Time in years between date of Fabry disease diagnosis and index date (continuous). If disease diagnosis occurred after index date, then the time difference is assumed to be missing.

^b^ The age at Fabry disease symptom onset was the lowest age at onset of any of the following Fabry disease symptoms: acroparesthesia, angiokeratomas, hypohidrosis, corneal whorling, gastrointestinal symptoms like diarrhea or abdominal pain, hearing loss, pulmonary changes, white matter lesions, and lymphedema.

^c^ The categorizations were developed programmatically by assessing the patients' DNA change and protein consequence using published literature and online databases [27, 28, 29, 30, 31] and resolving discrepancies manually.

^d^ Before matching, eGFR values were imputed.

**TABLE 2:** Study population for eGFR slope.

Characteristics	Before matching		After matching	
	Migalastat	ERT	Migalastat	ERT
*N*	268	119	222	222
Age at index, (years)				
Mean (SD)	51.7 (13.9)	46.3 (12.9)	50.3 (13.0)	50.7 (12.5)
Median (min, max)	54.0 (16.0, 78.0)	46.0 (18.0, 73.0)	52.0 (16.0, 75.0)	51.0 (18.0, 73.0)
Male sex, *n* (%)	142 (52.99%)	51 (42.86%)	108 (48.65%)	108 (48.65%)
Race, *n* (%)				
Asian	0 (0.00%)	1 (0.84%)	0 (0.00%)	0 (0.00%)
Black or African American	1 (0.37%)	1 (0.84%)	1 (0.45%)	4 (1.80%)
White	261 (97.39%)	107 (89.92%)	217 (97.75%)	203 (91.44%)
Not reported	6 (2.24%)	10 (8.40%)	4 (1.80%)	15 (6.76%)
ERT use, *n* (%)				
Agalsidase alfa	NA	46 (38.66%)	NA	73 (32.88%)
Agalsidase beta	NA	73 (61.34%)	NA	149 (67.12%)
Time since Fabry disease diagnosis^a^, years				
Mean (SD)	3.8 (5.3)	6.3 (9.7)	4.0 (5.5)	4.0 (7.2)
Median (min, max)	1.6 (0.0–35.3)	1.2 (0.0–49.0)	1.6 (0.0–35.3)	0.9 (0.0–47.9)
N missing	5	0	3	0
Age at onset of Fabry disease symptoms^b^, years				
Mean (SD)	42.2 (19.0)	26.3 (18.5)	42.2 (18.4)	28.8 (19.7)
Median (min, max)	47.0 (5.0, 74.0)	18.0 (10.0, 44.5)	46.0 (5.0, 74.0)	26.0 (3.0, 61.0)
N missing	99	20	80	53
Amenability, *n* (%)	268 (100.00%)	31 (26.05%)	222 (100.00%)	74 (33.33%)
Variant type^c^				
Frameshift variant	0 (0.00%)	16 (13.45%)	0 (0.00%)	30 (13.51%)
In‐frame deletion	2 (0.75%)	2 (1.68%)	2 (0.90%)	0 (0.00%)
In‐frame duplication	1 (0.37%)	0 (0.00%)	1 (0.45%)	0 (0.00%)
Initiation codon variant (start‐loss)	0 (0.00%)	2 (1.68%)	0 (0.00%)	3 (1.35%)
Large deletion (multi‐exon)	0 (0.00%)	3 (2.52%)	0 (0.00%)	6 (2.70%)
Missense variant	263 (98.13%)	80 (67.23%)	217 (97.75%)	143 (64.41%)
Nonsense variant (stop‐gain)	0 (0.00%)	11 (9.24%)	0 (0.00%)	20 (9.01%)
Splice site variant (canonical ±1/±2 or intronic)	0 (0.00%)	4 (3.36%)	0 (0.00%)	14 (6.31%)
Unclassified	2 (0.75%)	1 (0.84%)	2 (0.90%)	6 (2.70%)
eGFR, mL/min/1.73m^2^				
Mean (SD)	90.4 (23.9)	93.5 (21.5)	89.6 (20.1)	88.2 (18.6)
N missing	65	29	0^d^	0^d^
LVMI, g/m^2^				
Mean (SD)	109.9 (45.9)	119.9 (93.0)	106.1 (44.6)	121.2 (94.1)
Median (min, max)	96.4 (40.0–272.7)	98.1 (48.9–625.9)	93.4 (40.0–272.7)	97.6 (48.9–625.9)
N missing	172	79	140	139
Exposure time, years				
Mean (SD)	4.01 (1.51)	4.01 (1.54)	3.98 (1.52)	3.96 (1.52)
Median (min, max)	4.14 (0.91–6.99)	4.02 (1.00–6.97)	4.11 (0.91–6.99)	3.66 (1.00–6.97)
Sensitivity analysis exposure time, years				
Mean (SD)	—	—	3.83 (1.61)	3.81 (1.54)
Median (min, max)	—	—	3.83 (0.60–6.99)	3.66 (0.76–6.97)

Abbreviations: eGFR, estimated glomerular filtration rate; ERT, enzyme replacement therapy; LVMI, left ventricular mass index; max, maximum; min, minimum; NA, not applicable; SD, standard deviation.

^a^ Time in years between date of Fabry disease diagnosis and index date (continuous). If disease diagnosis occurred after index date, then the time difference is assumed to be missing.

^b^ The age at Fabry disease symptom onset was the lowest age at onset of any of the following Fabry disease symptoms: acroparesthesia, angiokeratomas, hypohidrosis, corneal whorling, gastrointestinal symptoms like diarrhea or abdominal pain, hearing loss, pulmonary changes, white matter lesions, and lymphedema.

^c^ The categorizations were developed programmatically by assessing the patients' DNA change and protein consequence using published literature and online databases [27, 28, 29, 30, 31] and resolving discrepancies manually.

^d^ Before matching, missing eGFR values were imputed.

### 3.2 Variables

Age at index, eGFR at index, and sex had a percent missingness of < 25% and were therefore included in the propensity score matching and in the statistical models. However, the additional variables considered for inclusion, including left ventricular mass index (LVMI), did not meet the criteria of < 25% missingness (Table S5) and were therefore not included in the PS matching and in the statistical models. Absolute SMDs for the matching variables age, sex, and eGFR were all < 0.1 after matching, and propensity scores were well distributed (Figure S1).

### 3.3 FACE Outcomes

The event rates and event rate ratios of FACEs using the Poisson model are shown in Table 3. For FACE event rates, migalastat treatment was numerically favored but not statistically significant vs. ERT for composite, cardiac, and renal FACEs; unadjusted cerebrovascular FACE rates with migalastat were statistically significantly lower than with ERT. Overall, patients experienced 55.3 composite FACEs/1000 patient‐years (95% CI: 40.7, 75.3) with migalastat and 100.7 (53.7, 188.9) with ERT; the event‐rate ratio was 0.55 (0.27, 1.11; *p* = 0.093). The event‐rate ratio for cerebrovascular FACEs was 0.24 (0.06, 0.88; unadjusted ***p* = 0.031**; BH‐adjusted *p* = 0.093), representing 4.7 FACEs/1000 patient‐years (2.0, 11.2) with migalastat and 19.8 (7.5, 52.4) with ERT. For cardiac FACEs, the event‐rate ratio was 0.61 (0.26, 1.45; unadjusted *p* = 0.266; BH‐adjusted *p* = 0.399), and for renal FACEs, the event‐rate ratio was 0.95 (0.10, 9.00; unadjusted and BH‐adjusted *p* = 0.964). The negative binomial model was evaluated due to over‐dispersed data, and results were similar (Table S6).

**TABLE 3:** Event rates for FACEs.

FACE	Events per 1000 person‐years (95% CI)		Event rate ratio (95% CI)	Unadjusted *p*‐value	BH‐adjusted *p*‐value
	Migalastat (*N* = 290)	ERT (*N* = 290)	Migalastat vs. ERT		
Composite	55.3 (40.7, 75.3)	100.7 (53.7, 188.9)	0.55 (0.27, 1.11)	0.093	—
Cardiac	47.8 (34.2, 66.8)	78.0 (35.2, 172.8)	0.61 (0.26, 1.45)	0.266	0.399
Cerebrovascular	4.7 (2.0, 11.2)	19.8 (7.5, 52.4)	0.24 (0.06, 0.88)	0.031	0.093
Renal	2.8 (0.9, 8.6)	3.0 (0.4, 20.9)	0.95 (0.10, 9.00)	0.964	0.964

*Note:* Event rate ratio < 1 favors migalastat.

Abbreviations: BH, Benjamini‐Hochberg; CI, confidence interval; ERT, enzyme replacement therapy; FACE, Fabry‐associated clinical event.

### 3.4 eGFR Outcomes

Results for eGFR slope for the main analysis are shown in Table 4. eGFR_CKD‐EPI_ slope was −1.47 mL/min/1.73m^2^/year (95% CI: −2.17, −0.77) with migalastat and −2.50 (−3.53, −1.46) with ERT. eGFR decline was numerically slower with migalastat vs. ERT treatment without reaching statistical significance, with a mean difference in the eGFR slopes of 1.03 (95% CI: −0.23 to 2.28; *p* = 0.109). The sensitivity analysis censoring visits after breaks of > 91 days showed similar results (Table S7).

**TABLE 4:** Annual eGFR slope (MMRM).

	Estimate (95% CI)	*p*
Migalastat	−1.47 (−2.17, −0.77)	—
ERT	−2.50 (−3.53, −1.46)	—
Difference	1.03 (−0.23, 2.28)	0.109

*Note:* Model including baseline eGFR, sex, and age.

Abbreviations: CI, confidence interval; eGFR, estimated glomerular filtration rate; ERT, enzyme replacement therapy; MMRM, mixed models with repeated measures.

### 3.5 Other Secondary Outcomes

Migalastat was numerically favored but not statistically significant vs. ERT for the proportion of patients with composite and cerebrovascular FACEs, with risk ratios of 0.83 (95% CI: 0.45, 1.52; *p* = 0.539) and 0.33 (95% CI: 0.09, 1.20; *p* = 0.092), respectively, while ERT was numerically favored but not statistically significant vs. migalastat for the proportion of patients with cardiac FACEs 1.09 (95% CI: 0.5, 2.34; *p* = 0.834), and there was no difference between migalastat and ERT for the proportion of patients with renal FACEs (Table S8).

For the incidence rate of FACEs assessed using the Poisson model, migalastat was numerically favored vs. ERT without reaching statistical significance for composite (*p* = 0.380), cerebrovascular (*p* = 0.075), and renal (*p* = 0.966) FACEs. For cardiac FACEs, migalastat and ERT were not different (Table S9).

Migalastat was also numerically favored vs. ERT without reaching statistical significance for all FACEs in the time to first FACE using the adjusted Cox proportional hazards model (all *p* > 0.05) (Table S10).

## 4 Discussion

This analysis compared FACE rates and annual eGFR slope in matched, previously treatment‐naïve patients with Fabry disease being initiated with either the oral small molecule chaperone migalastat or intravenous ERT enrolled in the followME Fabry registry. Propensity score matching with age, sex, and eGFR at baseline as matching variables was used to account for differences between patient groups. Poisson regression was used to analyze FACE rates, and MMRM was used to analyze eGFR slope.

Patients treated with migalastat showed a trend of fewer FACEs compared to ERT for composite, cardiac, renal, and cerebrovascular FACEs. Cerebrovascular FACE rates were statistically significantly lower only when not adjusting for multiple comparisons. Of note, the observed reduction in cerebrovascular FACEs, with an event‐rate ratio of 0.24 (95% CI 0.06, 0.88), is of a magnitude that is clinically meaningful, although it does not survive false discovery rate control across the outcome family of organ‐specific FACEs. In addition, there was a trend of slower eGFR decline with migalastat vs. ERT treatment. Migalastat was numerically favored vs. ERT (non‐significant) for the proportion of patients with composite and cerebrovascular FACEs (equivocal for renal FACEs), and ERT was marginally favored vs. migalastat (non‐significant) for the proportion of cardiac FACEs. Migalastat was favored without reaching statistical significance for the incidence of composite, cerebrovascular, and renal FACEs (equivocal for incidence of cardiac FACEs), and favored (non‐significant) for all FACEs in the time to first FACE analysis.

Several studies have been conducted in Fabry disease evaluating or comparing different ERTs using real‐world evidence. For example, Fabry patients treated with ERT in the Canadian Fabry disease initiative were followed for 5 years for clinical outcomes including death, neurologic and cardiovascular events, and kidney function. The study found that targeted use of ERT in patients who met ERT criteria, along with cardiovascular risk factor modification, reduced Fabry‐related adverse outcomes [32]. A multi‐center, retrospective cohort study compared clinical outcomes and biochemical response in patients with Fabry treated with agalsidase alfa vs. beta. No difference in clinical event rate was found between the two treatments. However, patients treated with agalsidase beta had a greater decrease in Gb3 and better reduction in left ventricular mass [33]. Another recent study evaluated the incidence and outcome of stroke in patients with Fabry disease treated with agalsidase beta vs. untreated patients using global data from the Fabry registry. This study found that strokes are common in Fabry disease and treatment with agalsidase beta lowered stroke risk [24].

Previous clinical trial and real‐world studies have shown comparable results with migalastat. In an integrated long‐term post hoc analysis of all patients in the Phase 3 clinical trial and open label extension population, patients treated with migalastat showed stable renal function [14] and low FACE incidence and prevalence [21] overall and in both ERT‐naïve and ERT‐experienced cohorts over ~5 years. These findings were consistent with those in an analysis of patients in the followME Fabry registry receiving migalastat for ~4 years [18]. Compared to that report, migalastat‐treated patients included in the current analysis are younger due to the requirement of being treatment‐naïve [18]. Additionally, the Phase 3 ATTRACT study, in ERT‐experienced patients with amenable variants, was a randomized study comparing ERT to migalastat over 18 months, which showed numerically fewer FACEs in the migalastat arm and similar eGFR slope to the current study [12]. The 12‐month open‐label extension of ATTRACT showed similar results [13] as did a post hoc analysis of several Phase 3 clinical studies [21].

The studies described above show the effectiveness of ERT and migalastat on clinical outcomes in patients with Fabry disease. In the current study, we demonstrated that treatment with oral migalastat resulted in numerically fewer composite, cardiac, and renal FACEs compared to ERT, despite being statistically not significant. Notably, given the data showing that strokes are common in patients with Fabry disease [24], there was a statistically significantly lower unadjusted event rate for cerebrovascular FACEs with migalastat vs. ERT in this analysis. One hypothesis is that the observed difference in cerebrovascular FACEs may be partly attributable to the ability of migalastat, as a low‐molecular‐weight small molecule, to penetrate the central nervous system, a property demonstrated in preclinical studies [34], in contrast to intravenously administered ERT, whose large molecular size limits blood–brain barrier penetration. Whether this translates into clinical benefit on cerebrovascular outcomes in humans warrants further investigation. In addition, patients treated with migalastat had a trend toward preservation of eGFR vs. those treated with ERT. Together these data show the importance of these types of comparative effectiveness analyses in Fabry disease and may suggest clinically relevant differences between therapeutic options.

This study has several strengths. Real‐world data from a prospective registry database matched a large cohort of patients with Fabry disease for this analysis. Groups were well matched and exposure to treatment was similar between migalastat and ERT, allowing for a robust comparison. These data, demonstrating the comparative effectiveness of migalastat vs. ERT, address a clinical evidence gap in a broader and more representative Fabry disease population treated under routine clinical practice conditions complementary to the evidence from randomized clinical trials alone.

The study also has some limitations. Patient matching did not include variant amenability as a matching variable. Because frameshift, null, and structural variants are not amenable, it is possible that these and other severe types of variants are present in the ERT group and would not be present in the migalastat group. This is a possible source of imbalance between the groups. This reflects the real‐world clinical practice: in the followME Fabry registry, only approximately 25% of ERT‐treated patients carried an amenable variant, since patients with amenable variants are more commonly initiated on migalastat in routine practice. Restricting the matched analysis to amenable ERT patients only was explored but is not considered a methodologically appropriate sensitivity analysis for several reasons. The number of ERT patients with amenable variants is small (*n* = 36 for the FACE outcomes analysis; *n* = 31 for the eGFR slope analysis). Matching e.g., 230 migalastat patients in the FACE outcomes analysis to only 36 amenable ERT patients on a 1:1 basis with replacement results in a median repetition of approximately seven times per ERT patient. Indeed, when this matching is performed, 31% of the patients are repeated ≥ 10 times, while only 2% of the patients were repeated ≥ 10 times in the FACE outcomes analysis when non‐amenable ERT patients are included (Table S2). In contrast, in the main analysis, 64% of ERT patients are used only once or twice, preserving the statistical properties of the matched cohort, versus only 19% in an analysis restricted to amenable ERT patients. The shift toward extreme repetition in an amenable‐only analysis changes the distributional shape of propensity scores and concentrates influence on a very small number of individual patients, rendering the resulting estimates unreliable and susceptible to large uncertainty. It should therefore be acknowledged that, since treatment groups were not matched on amenability status, a contribution of genetic differences to the observed outcome differences cannot be excluded. Further variables that would ideally be used for matching, such as LVMI and proteinuria, had > 25% missingness, or were not formally reported by the registry (Fabry phenotype classic vs. non‐classic), and were therefore not used for propensity score matching. Some of the Fabry disease symptoms such as corneal whorling were not well balanced after matching. However, patients were matched based on important predictive markers including eGFR, age and gender to allow for a comparison balanced for disease severity. Age, gender and eGFR have previously been shown to be predictors of clinical outcomes [14, 21, 35, 36, 37]. All patients included in this analysis were treatment‐naïve and had to meet eligibility according to local guidelines for treatment initiation implying similar disease burden in both groups. Moreover, missense variants prevail in both treatment groups (Tables 1 and 2). Missense variants are more commonly associated with residual α‐gal‐A enzyme activity and later‐onset phenotypes compared to null variants [38], suggesting the overall analysis population is unlikely to be predominantly classic Fabry. Recent kidney disease guidelines have placed greater emphasis on cystatin C‐based assessment of kidney function, reflecting growing recognition of the limitations of creatinine alone in patients with altered muscle mass [39]. Cystatin C was not collected in followME and could therefore not be used for eGFR calculation; however, the use of a more sensitive marker, such as cystatin C, may show greater differences in kidney disease progression. Nevertheless, creatinine‐based eGFR remains the most widely used renal function measure in routine practice and clinical studies, and longitudinal changes in creatinine‐based eGFR continue to provide clinically meaningful information regarding kidney disease progression [40]. Residual α‐gal‐A enzyme activity was not routinely collected in followME and was therefore unavailable for analysis. Residual enzyme activity is an important disease characteristic in males with Fabry disease and may differ across *GLA* genotypes. As a result, the possibility that differences in residual enzyme activity contributed to observed outcomes cannot be excluded. This should be considered alongside the broader genotypic and amenability limitations discussed above.

In summary, this comprehensive analysis of the real‐world effectiveness of migalastat vs. ERT supports the multisystemic effectiveness of chaperone therapy as measured by FACE and eGFR decline rates in Fabry disease. Comparisons numerically favored migalastat over ERT for the primary FACE outcomes, for eGFR slope and most of the other secondary outcomes. ERT was marginally favored vs. migalastat only for the proportion of patients with cardiac FACEs, with a difference of less than one percentage point that was not statistically significant.

The followME Fabry Pathfinders registry is continuing to collect data on Fabry disease treatments which will provide an opportunity for future analyses, e.g., covering LVMI as an important cardiac measure for Fabry disease patients, and potentially assessing the impact of amenable variants on FACEs.

## Author Contributions

Conceptualization: Alasdair MacCulloch, Biliana O. Veleva‐Rotse, Vera Gielen. Data curation: Agnieszka Kopiec. Formal analysis: Agnieszka Kopiec, Noemi Hummel. Funding acquisition: Alasdair MacCulloch. Investigation: Peter Nordbeck. Methodology: Noemi Hummel. Supervision: Alasdair MacCulloch, Biliana O. Veleva‐Rotse. Visualization: Agnieszka Kopiec. Writing – original draft: Agnieszka Kopiec, Noemi Hummel. Writing – review and editing: Alasdair MacCulloch, Agnieszka Kopiec, Biliana O. Veleva‐Rotse, Noemi Hummel, Peter Nordbeck, Vera Gielen.

## Funding

The study was funded by Amicus Therapeutics UK Ltd. As of April 27, 2026, Amicus is a wholly owned subsidiary of BioMarin Pharmaceutical Inc. Project management support and submission assistance were provided by Amiculum and funded by Amicus Therapeutics UK Ltd.

## Ethics Statement

The followME Fabry Pathfinders study was conducted in accordance with the ethical principles of the Declaration of Helsinki and Good Pharmacoepidemiology Practice guidelines, along with applicable privacy laws and local regulations for each participating site. All patients provided written informed consent for participation in this study.

## Conflicts of Interest

A.M., B.O.V.‐R., and V.G. are employees of BioMarin Pharmaceutical Inc. A.K. and N.H. are employees of Certara, which is a paid consultancy from Amicus Therapeutics. P.N. reports consultancy/advisory board fees from Amicus Therapeutics, Chiesi, Idorsia, Sangamo, Sanofi and Takeda; and speakers' bureau/honoraria for non‐CME activities from Amicus Therapeutics, Chiesi, Idorsia, Sangamo, Sanofi and Takeda.

## Supporting information


**Table S1:** Inclusion and exclusion criteria.
**Table S2:** Weights of ERT patients.
**Table S3:** Additional baseline characteristics: Study population for FACE outcomes.
**Table S4:** Additional baseline characteristics: Study population for eGFR outcomes.
**Table S5:** followME registry variable missingness.
**Table S6:** Event rates for FACEs (negative binomial model).
**Table S7:** Annual eGFR slope (MMRM, sensitivity analysis).
**Table S8:** Proportion of patients with FACEs.
**Table S9:** Incidence rate of FACEs.
**Table S10:** Time to first FACE.
**Figure S1:** Standardized mean differences and propensity score distributions.

## Data Availability

The de‐identified individual participant data that underlie the results reported in this article (including text, tables, figures, and appendices) will be made available together with the research protocol and data dictionaries, for non‐commercial, academic purposes. Additional supporting documents may be available upon request. For more information see BioMarin Data Sharing Web Page at https://www.biomarin.com/publication‐data‐request/.

## References

[1] D. P. Germain , “Fabry Disease,” Orphanet Journal of Rare Diseases 5 (2010): 30. 10.1186/1750-1172-5-30 PMC3009617 21092187

[2] S. Waldek , M. R. Patel , M. Banikazemi , R. Lemay , and P. Lee , “Life Expectancy and Cause of Death in Males and Females With Fabry Disease: Findings From the Fabry Registry,” Genetics in Medicine 11, no. 11 (2009): 790–796. 10.1097/GIM.0b013e3181bb05bb 19745746

[3] A. Ortiz , D. P. Germain , R. J. Desnick , et al., “Fabry Disease Revisited: Management and Treatment Recommendations for Adult Patients,” Molecular Genetics and Metabolism 123, no. 4 (2018): 416–427. 10.1016/j.ymgme.2018.02.014 29530533

[4] R. J. Hopkin , D. Laney , S. Kazemi , and A. Walter , “Fabry Disease in Females: Organ Involvement and Clinical Outcomes Compared With the General Population,” Orphanet Journal of Rare Diseases 20, no. 1 (2025): 433. 10.1186/s13023-025-03922-x PMC12351852 40804726

[5] S. Kallish , A. Camporeale , R. J. Hopkin , et al., “Long‐Term Efficacy of Migalastat in Females With Fabry Disease,” Journal of Medical Genetics 62, no. 12 (2025): 798–807. 10.1136/jmg-2024-110596 PMC12703307 40897525

[6] U. Ramaswami , G. Pintos‐Morell , C. Kampmann , et al., “Two Decades of Experience of the Fabry Outcome Survey Provides Further Confirmation of the Long‐Term Effectiveness of Agalsidase Alfa Enzyme Replacement Therapy,” Molecular Genetics and Metabolism Reports 43 (2025): 101215. 10.1016/j.ymgmr.2025.101215 PMC12018052 40276560

[7] C. Wanner , A. Ortiz , W. R. Wilcox , et al., “Global Reach of Over 20 Years of Experience in the Patient‐Centered Fabry Registry: Advancement of Fabry Disease Expertise and Dissemination of Real‐World Evidence to the Fabry Community,” Molecular Genetics and Metabolism 139, no. 3 (2023): 107603. 10.1016/j.ymgme.2023.107603 37236007

[8] M. Zhang and C. Wang , “Therapeutic Landscape of Fabry Disease: Advances and Challenges From Classical Strategies to Emerging Therapies,” Frontiers in Medicine (Lausanne) 12 (2025): 1662867. 10.3389/fmed.2025.1662867 PMC12616745 41244789

[9] M. Lenders , F. Stappers , and E. Brand , “In Vitro and In Vivo Amenability to Migalastat in Fabry Disease,” Mol Ther Methods Clin Dev 19 (2020): 24–34. 10.1016/j.omtm.2020.08.012 PMC7490640 32995357

[10] E. R. Benjamin , M. C. Della Valle , X. Wu , et al., “The Validation of Pharmacogenetics for the Identification of Fabry Patients to Be Treated With Migalastat,” Genetics in Medicine : Official Journal of the American College of Medical Genetics 19, no. 4 (2017): 430–438. 10.1038/gim.2016.122 PMC5392595 27657681

[11] D. P. Germain , D. A. Hughes , K. Nicholls , et al., “Treatment of Fabry's Disease With the Pharmacologic Chaperone Migalastat,” New England Journal of Medicine 375, no. 6 (2016): 545–555. 10.1056/NEJMoa1510198 27509102

[12] D. A. Hughes , K. Nicholls , S. P. Shankar , et al., “Oral Pharmacological Chaperone Migalastat Compared With Enzyme Replacement Therapy in Fabry Disease: 18‐Month Results From the Randomised Phase III ATTRACT Study,” Journal of Medical Genetics 54, no. 4 (2017): 288–296. 10.1136/jmedgenet-2016-104178 PMC5502308 27834756

[13] U. Feldt‐Rasmussen , D. Hughes , G. Sunder‐Plassmann , et al., “Long‐Term Efficacy and Safety of Migalastat Treatment in Fabry Disease: 30‐Month Results From the Open‐Label Extension of the Randomized, Phase 3 ATTRACT Study,” Molecular Genetics and Metabolism 131, no. 1–2 (2020): 219–228. 10.1016/j.ymgme.2020.07.007 33012654

[14] D. G. Bichet , R. Torra , E. Wallace , et al., “Long‐Term Follow‐Up of Renal Function in Patients Treated With Migalastat for Fabry Disease,” Molecular Genetics and Metabolism Reports 28 (2021): 100786. 10.1016/j.ymgmr.2021.100786 PMC8353473 34401344

[15] M. Lenders , P. Nordbeck , C. Kurschat , et al., “Treatment of Fabry Disease Management With Migalastat‐Outcome From a Prospective 24 Months Observational Multicenter Study (FAMOUS),” European Heart Journal ‐ Cardiovascular Pharmacotherapy 8, no. 3 (2022): 272–281. 10.1093/ehjcvp/pvab025 35512362

[16] M. Lenders , P. Nordbeck , C. Kurschat , et al., “Treatment of Fabry's Disease With Migalastat: Outcome From a Prospective Observational Multicenter Study (FAMOUS),” Clinical Pharmacology and Therapeutics 108, no. 2 (2020): 326–337. 10.1002/cpt.1832 32198894

[17] J. Müntze , D. Gensler , O. Maniuc , et al., “Oral Chaperone Therapy Migalastat for Treating Fabry Disease: Enzymatic Response and Serum Biomarker Changes After 1 Year,” Clinical Pharmacology and Therapeutics 105, no. 5 (2019): 1224–1233. 10.1002/cpt.1321 PMC6590383 30506669

[18] D. A. Hughes , G. Sunder‐Plassmann , A. Jovanovic , et al., “Renal and Multisystem Effectiveness of 3.9 Years of Migalastat in a Global Real‐World Cohort: Results From the followME Fabry Pathfinders Registry,” Journal of Inherited Metabolic Disease 48, no. 1 (2025): e12771. 10.1002/jimd.12771 PMC11730455 39031114

[19] European Network of Centres for Pharmacoepidemiology and Pharmacovigilance , “A Prospective, Observational Registry of Patients With Fabry Disease,” accessed October 16, 2025, https://catalogues.ema.europa.eu/node/2303/administrative‐details.

[20] A. S. Levey , L. A. Stevens , C. H. Schmid , et al., “A New Equation to Estimate Glomerular Filtration Rate,” Annals of Internal Medicine 150, no. 9 (2009): 604–612. 10.7326/0003-4819-150-9-200905050-00006 PMC2763564 19414839

[21] D. A. Hughes , D. G. Bichet , R. Giugliani , et al., “Long‐Term Multisystemic Efficacy of Migalastat on Fabry‐Associated Clinical Events, Including Renal, Cardiac and Cerebrovascular Outcomes,” Journal of Medical Genetics 60, no. 7 (2023): 722–731. 10.1136/jmg-2022-108669 PMC10359570 36543533

[22] S.‐C. Chow , J. Shao , H. Wang , and Y. Lokhnygina , Sample Size Calculations in Clinical Research, 3rd ed. (Chapman and Hall/CRC, 2017).

[23] K. Azimpour , C. Tordoff‐Gibson , P. Dorling , I. Koulinska , V. Laliman‐Khara , and A. Forsythe , “EE64 Impact of Baseline Characteristics on Indirect Treatment Comparisons (ITCS) in Fabry Disease: A Systematic Literature Review (SLR),” Value in Health 26, no. 6 (2023): S71.

[24] A. Burlina , L. al‐Shaar , D. P. Germain , et al., “Reduced Incidence of Stroke in Patients With Fabry Disease Treated With Agalsidase Beta: A Matched Analysis From the Fabry Registry,” Molecular Genetics and Metabolism 144, no. 2 (2025): 108669.

[25] Y. Benjamini and Y. Hochberg , “Controlling the False Discovery Rate: A Practical and Powerful Approach to Multiple Testing,” Journal of the Royal Statistical Society, Series B: Statistical Methodology 57, no. 1 (1995): 289–300.

[26] Y. Benjamini and D. Yekutieli , “The Control of the False Discovery Rate in Multiple Testing Under Dependency,” Annals of Statistics 29, no. 4 (2001): 1165–1188.

[27] S. Richards , N. Aziz , S. Bale , et al., “Standards and Guidelines for the Interpretation of Sequence Variants: A Joint Consensus Recommendation of the American College of Medical Genetics and Genomics and the Association for Molecular Pathology,” Genetics in Medicine 17, no. 5 (2015): 405–424. 10.1038/gim.2015.30 PMC4544753 25741868

[28] HGVS Nomenclature (Human Genome Variation Society) , “HGVS Nomenclature,” accessed June 9, 2026, https://hgvs‐nomenclature.org/stable/.

[29] J. T. den Dunnen , R. Dalgleish , D. R. Maglott , et al., “HGVS Recommendations for the Description of Sequence Variants: 2016 Update,” Human Mutation 37, no. 6 (2016): 564–569. 10.1002/humu.22981 26931183

[30] R. K. Hart , I. F. A. C. Fokkema , M. DiStefano , et al., “HGVS Nomenclature 2024: Improvements to Community Engagement, Usability, and Computability,” Genome Medicine 16, no. 1 (2024): 149. 10.1186/s13073-024-01421-5 PMC11660784 39702242

[31] ClinGen Sequence Variant Interpretation (SVI) Working Group , “*ClinGen Variant Classification Guidance*,” accessed June 9, 2026, https://www.clinicalgenome.org/tools/clingen‐variant‐classification‐guidance/.

[32] S. M. Sirrs , D. G. Bichet , R. Casey , et al., “Outcomes of Patients Treated Through the Canadian Fabry Disease Initiative,” Molecular Genetics and Metabolism 111, no. 4 (2014): 499–506. 10.1016/j.ymgme.2014.01.014 24534763

[33] M. Arends , M. Biegstraaten , C. Wanner , et al., “Agalsidase Alfa Versus Agalsidase Beta for the Treatment of Fabry Disease: An International Cohort Study,” Journal of Medical Genetics 55, no. 5 (2018): 351–358. 10.1136/jmedgenet-2017-104863 PMC5931248 29437868

[34] Y. S. Wu , R. Khanna , V. Schmith , et al., “Migalastat Tissue Distribution: Extrapolation From Mice to Humans Using Pharmacokinetic Modeling and Comparison With Agalsidase Beta Tissue Distribution in Mice,” Clinical Pharmacology in Drug Development 10, no. 9 (2021): 1075–1088. 10.1002/cpdd.941 PMC8453552 33876577

[35] D. G. Bichet , J. M. Aerts , C. Auray‐Blais , et al., “Assessment of Plasma Lyso‐Gb(3) for Clinical Monitoring of Treatment Response in Migalastat‐Treated Patients With Fabry Disease,” Genetics in Medicine 23, no. 1 (2021): 192–201. 10.1038/s41436-020-00968-z PMC7790748 32994552

[36] C. Wanner , J. P. Oliveira , A. Ortiz , et al., “Prognostic Indicators of Renal Disease Progression in Adults With Fabry Disease: Natural History Data From the Fabry Registry,” Clinical Journal of the American Society of Nephrology 5, no. 12 (2010): 2220–2228. 10.2215/CJN.04340510 PMC2994083 20813854

[37] B. Haycroft , A. Stevenson , R. Stork , et al., “Targeted Literature Review Exploring the Predictive Value of Estimated Glomerular Filtration Rate and Left Ventricular Mass Index as Indicators of Clinical Events in Fabry Disease,” Orphanet Journal of Rare Diseases 18, no. 1 (2023): 322. 10.1186/s13023-023-02936-7 PMC10571251 37828551

[38] A. Mehta and D. A. Hughes , “Fabry Disease,” in GeneReviews, ed. M. P. Adam , S. Bick , G. M. Mirzaa , S. E. Wallace , and A. Amemiya (University of Washington, Seattle, 2024). 20301469

[39] KDIGO 2024 , “Clinical Practice Guideline for the Evaluation and Management of Chronic Kidney Disease,” Kidney International 105, no. 4s (2024): S117–s314. 10.1016/j.kint.2023.10.018 38490803

[40] E. R. Gottlieb , C. Estiverne , N. V. Tolan , S. E. F. Melanson , and M. L. Mendu , “Estimated GFR With Cystatin C and Creatinine in Clinical Practice: A Retrospective Cohort Study,” Kidney Medicine 5, no. 3 (2023): 100600. 10.1016/j.xkme.2023.100600 PMC9984886 36879723

