# Supplementary material for: Migalastat Versus Enzyme Replacement Therapy in Treatment‐Naïve Fabry Disease: Real‐World Evidence From the followME Fabry Pathfinders Registry
Source: J Inherit Metab Dis. 2026 Nov 9;49(6):e70261. doi: 10.1002/jimd.70261 (PMC13747706; doi:10.1002/jimd.70261)
Supplement: Supplementary file 1 [file JIMD-49-0-s001.docx]

Migalastat Versus Enzyme Replacement Therapy in Treatment-Naïve Fabry Disease: Real-world Evidence from the followME Fabry Pathfinders Registry

# Supplementary Material

Peter Nordbeck, Agnieszka Kopiec, Noemi Hummel, Vera Gielen, Biliana O Veleva-Rotse, Alasdair MacCulloch

## Table S1 | Inclusion and exclusion criteria

| **Inclusion criteria** | **Exclusion criteria** |
| --- | --- |
| 1. followME Fabry registry inclusion criteria:    a. Confirmed diagnosis of Fabry disease    b. eGFR >30 mL/min/1.73 m^2^ at enrolment 2. ≥16 years of age 3. Initiated first treatment in the 24 months prior to enrollment or any time after enrollment without prior treatment 4. If treated with migalastat: have amenable GLA gene variants 5. For eGFR slope analysis: have ≥3 eGFR assessments, including the baseline assessment | 1. Participated in a clinical trial of any investigational product or device at the time of enrollment 2. Failed to meet eligibility requirements 3. Had a protocol violation 4. Withdrew at enrollment |

## Table S2 │ Weights of ERT patients.

| **Weight** | **Frequency of ERT patients in the FACE outcomes population** | **Frequency of ERT patients in the eGFR slope population** |
| --- | --- | --- |
| 1 | 40 | 20 |
| 2 | 28 | 24 |
| 3 | 11 | 15 |
| 4 | 12 | 8 |
| 5 | 5 | 5 |
| 6 | 2 | 5 |
| 7 | 4 | 2 |
| 8 | - | 1 |
| 9 | 2 | - |
| 10 | 1 | - |
| 20 | 1 | - |
| **Weighted sum** | **290** | **222** |

Abbreviations: eGFR, estimated glomerular filtration rate; ERT, enzyme replacement therapy; FACE, Fabry-associated clinical event.

## Table S3 │ Additional baseline characteristics: Study population for FACE outcomes.

|  | **Before matching** | | **After matching** | |
| --- | --- | --- | --- | --- |
| **Characteristics** | **Migalastat** | **ERT** | **Migalastat** | **ERT** |
| **N** | 319 | 140 | 290 | 290 |
| **Weight, kg** | | | | |
| Mean (SD) | 78.1 (15.9) | 76.2 (12.8) | 78.4 (16.1) | 76.7 (12.5) |
| Median (Min, Max) | 77.1 (45.0 - 135.0) | 76.0 (49.0 - 105.0) | 77.4 (45.0 - 135.0) | 78.5 (49.0 - 105.0) |
| N missing (%) | 121 (37.93%) | 48 (34.29%) | 110 (37.93%) | 115 (39.66%) |
| **BMI, kg/m^2^** | | | | |
| Mean (SD) | 26.8 (4.9) | 27.0 (5.1) | 26.9 (4.9) | 27.0 (4.8) |
| Median (Min, Max) | 26.0 (16.0 - 42.0) | 27.0 (17.0 - 41.0) | 26.0 (16.0 - 42.0) | 28.0 (17.0 - 41.0) |
| N missing (%) | 133 (41.69%) | 53 (37.86%) | 121 (41.72%) | 126 (43.45%) |
| **Fabry symptoms, n (%)** | | | | |
| Corneal whorling | 35 (10.97%) | 63 (45.00%) | 34 (11.72%) | 106 (36.55%) |
| Angiokeratomas | 41 (12.85%) | 56 (40.00%) | 37 (12.76%) | 87 (30.00%) |
| Acroparesthesia | 77 (24.14%) | 82 (58.57%) | 74 (25.52%) | 128 (44.14%) |
| Hypohidrosis | 34 (10.66%) | 48 (34.29%) | 29 (10.00%) | 74 (25.52%) |
| Gastrointestinal symptoms | 72 (22.57%) | 54 (38.57%) | 65 (22.41%) | 88 (30.34%) |
| Hearing loss | 73 (22.88%) | 29 (20.71%) | 67 (23.10%) | 62 (21.38%) |
| Brain MRI changes (white matter lesions) | 67 (21.00%) | 33 (23.57%) | 66 (22.76%) | 77 (26.55%) |
| Lymphedema | 11 (3.45%) | 11 (7.86%) | 11 (3.79%) | 16 (5.52%) |
| Pulmonary changes | 23 (7.21%) | 28 (20.00%) | 20 (6.90%) | 48 (16.55%) |
| Diabetes | 19 (5.96%) | 5 (3.57%) | 17 (5.86%) | 15 (5.17%) |
| Dyslipidemia | 111 (34.80%) | 50 (35.71%) | 103 (35.52%) | 106 (36.55%) |
| Hypertension | 138 (43.26%) | 56 (40.00%) | 124 (42.76%) | 141 (48.62%) |
| Abbreviations: ERT, enzyme replacement therapy; Min, minimum; Max, maximum; MRI, magnetic resonance imaging; SD, standard deviation. | | | | |

## Table S4 │ Additional baseline characteristics: Study population for eGFR outcomes.

|  | **Before matching** | | **After matching** | |
| --- | --- | --- | --- | --- |
| **Characteristics** | **Migalastat** | **ERT** | **Migalastat** | **ERT** |
| **N** | 268 | 119 | 222 | 222 |
| **Weight, kg** | | | | |
| Mean (SD) | 78.5 (15.3) | 76.5 (12.2) | 78.5 (15.8) | 78.7 (12.8) |
| Median (Min, Max) | 77.4 (50.0 - 135.0) | 77.0 (54.0 - 103.0) | 77.1 (50.0 - 135.0) | 80.0 (54.0 - 103.0) |
| N missing (%) | 108 (40.30%) | 42 (35.29%) | 88 (39.64%) | 74 (33.33%) |
| **BMI, kg/m^2^** | | | | |
| Mean (SD) | 26.9 (4.6) | 27.4 (5.3) | 26.9 (4.7) | 27.8 (5.2) |
| Median (Min, Max) | 26.0 (16.0 - 42.0) | 27.0 (17.0 - 41.0) | 26.0 (16.0 - 42.0) | 27.0 (18.0 - 41.0) |
| N missing (%) | 118 (44.03%) | 47 (39.50%) | 97 (43.69%) | 76 (34.23%) |
| **Fabry symptoms, n (%)** | | | | |
| Corneal whorling | 32 (11.94%) | 56 (47.06%) | 27 (12.16%) | 86 (38.74%) |
| Angiokeratomas | 35 (13.06%) | 46 (38.66%) | 29 (13.06%) | 74 (33.33%) |
| Acroparesthesia | 68 (25.37%) | 67 (56.30%) | 55 (24.77%) | 113 (50.90%) |
| Hypohidrosis | 30 (11.19%) | 41 (34.45%) | 21 (9.46%) | 60 (27.03%) |
| Gastrointestinal symptoms | 62 (23.13%) | 45 (37.82%) | 48 (21.62%) | 73 (32.88%) |
| Hearing loss | 64 (23.88%) | 27 (22.69%) | 49 (22.07%) | 51 (22.97%) |
| Brain MRI changes (white matter lesions) | 63 (23.51%) | 32 (26.89%) | 59 (26.58%) | 58 (26.13%) |
| Lymphedema | 11 (4.10%) | 11 (9.24%) | 9 (4.05%) | 17 (7.66%) |
| Pulmonary changes | 21 (7.84%) | 25 (21.01%) | 15 (6.76%) | 43 (19.37%) |
| Diabetes | 16 (5.97%) | 5 (4.20%) | 15 (6.76%) | 15 (6.76%) |
| Dyslipidemia | 98 (36.57%) | 46 (38.66%) | 81 (36.49%) | 86 (38.74%) |
| Hypertension | 119 (44.40%) | 53 (44.54%) | 122 (54.95%) | 109 (49.10%) |
| Abbreviations: ERT, enzyme replacement therapy; Min, minimum; Max, maximum; MRI, magnetic resonance imaging; SD, standard deviation. | | | | |

## Table S5 │ followME registry variable missingness.

| **Variable** | **Missingness, n/N (%)** |
| --- | --- |
| eGFR at index | 101/459 and 94/387 (22.0-24.3) |
| LVMI at index | 292/459 and 251/387 (63.6-64.9) |
| Urine protein to creatine ratio at index | 266/459 and 223/387 (57.6-58.0) |
| Angiotensin II receptor blocker or ACE inhibitor use at index | Unable to distinguish between no ACEi/ARB and missing data^a^ |
| History of FACEs | Unable to distinguish between no FACE and missing data^a^ |
| ^a^ Concomitant medications and history of FACEs reporting was not mandatory in followME.  Abbreviations: ACE, angiotensin converting enzyme; eGFR, estimated glomerular filtration rate; FACE, Fabry-associated clinical event; LVMI, left ventricular mass index; NA, not applicable. | |

## Table S6 │Event rates for FACEs (negative binomial model).

|  | **Events per 1000 person-years (95% CI)** | | **Event rate ratio (95% CI)** | **Unadjusted**  **p-value** |
| --- | --- | --- | --- | --- |
|  | **Migalastat (N=290)** | **ERT (N=290)** | **Migalastat vs ERT** |  |
| **Composite FACE** | 55.0 (40.2, 75.1) | 92.1 (49.8, 170.6) | 0.60 (0.30, 1.19) | 0.143 |
| **Cardiac FACE** | 47.4 (33.6, 66.9) | 69.7 (32.0, 151.8) | 0.68 (0.29, 1.59) | 0.376 |
| **Cerebrovascular FACE** | 4.8 (2.0, 11.4) | 19.3 (7.4, 50.8) | **0.25 (0.07, 0.91)** | **0.035** |
| **Renal FACE** | 2.8 (0.9, 8.6) | 3.0 (0.4, 20.9) | 0.95 (0.10, 9.01) | 0.964 |
| Event rate ratio < 1 favors MIG.  Bold indicates statistical significance.  Abbreviations: CI, confidence interval; ERT, enzyme replacement therapy; FACE, Fabry-associated clinical event. | | | | |

## Table S7 │Annual eGFR slope (MMRM, sensitivity analysis).

|  | **Estimate** | **95% CI** | **P-value** |
| --- | --- | --- | --- |
| **Migalastat** | -1.47 | -2.21, -0.74 | - |
| **ERT** | -2.47 | -3.53, -1.41 | - |
| **Difference** | 0.99 | -0.29, 2.28 | 0.1301 |
| Model including baseline eGFR, sex, and age.  Abbreviations: CI, confidence interval; eGFR, estimated glomerular filtration rate; ERT, enzyme replacement therapy; MMRM, mixed models with repeated measures. | | | |

## Table S8 │ Proportion of patients with FACEs.

| **FACE** | **Proportion of patients with FACES, % (95% CI)** | | **Risk ratio (95% CI)** | **p-value** |
| --- | --- | --- | --- | --- |
|  | **Migalastat (N=290)** | **ERT (N=290)** | **Migalastat vs ERT** |  |
| **Composite** | 14.8 (11.3, 19.5) | 17.9 (10.5, 30.8) | 0.83 (0.45, 1.52)) | 0.539 |
| **Cardiac** | 13.1 (9.7, 17.6) | 12.1 (5.9, 24.5) | 1.09 (0.50, 2.34) | 0.834 |
| **Cerebrovascular** | 1.7 (0.7, 4.1) | 5.2 (2.0, 13.2) | 0.33 (0.09, 1.20) | 0.092 |
| **Renal** | 1.0 (0.3, 3.2) | 1.0 (0.2, 7.3) | 1.00 (0.10, 9.56) | 1.000 |
| Risk ratio < 1 favors migalastat.  Abbreviations: CI, confidence interval; ERT: enzyme replacement therapy; FACE: Fabry-associated clinical event. | | | | |

## Table S9 │ Incidence rate of FACEs.

| **FACE** | **Incidence per 1000 person-years, (95% CI)** | | **Incidence rate ratio (95% CI)** | **p-value** |
| --- | --- | --- | --- | --- |
|  | **Migalastat (N=290)** | **ERT (N=290)** | **Migalastat vs ERT** |  |
| **Composite** | 44.38 (33.0, 59.8) | 59.9 (32.9, 109.2) | 0.74 (0.38, 1.45) | 0.380 |
| **Cardiac** | 38.9 (28.3, 53.4) | 38.8 (18.0, 84.0) | 1.00 (0.43, 2.31) | 0.997 |
| **Cerebrovascular** | 4.7 (2.0, 11.3) | 15.3 (5.9, 40.0) | 0.31 (0.08, 1.12) | 0.075 |
| **Renal** | 2.8 (0.9, 8.7) | 3.0 (0.4, 21.0) | 0.95 (0.10, 9.08) | 0.966 |
| Incidence risk ratio < 1 favors migalastat.  Abbreviations: CI, confidence interval; ERT, enzyme replacement therapy; FACE, Fabry-associated clinical event. | | | | |

## Table S10 │ Time to first FACE.

| **FACE** | **Unadjusted Cox model** | | **Adjusted Cox model** | |
| --- | --- | --- | --- | --- |
|  | **HR migalastat vs ERT (95% CI)** | **p-value** | **HR migalastat vs ERT (95% CI)** | **p-value** |
| **Composite** | 0.74 (0.39, 1.42) | 0.363 | 0.66 (0.33, 1.34) | 0.249 |
| **Cardiac** | 1.01 (0.46, 2.25) | 0.975 | 0.91 (0.39, 2.13) | 0.824 |
| **Cerebrovascular** | 0.31 (0.08, 1.13) | 0.076 | 0.29 (0.08, 1.10) | 0.068 |
| **Renal** | 0.84 (0.09, 7.90) | 0.875 | 0.75 (0.14, 4.22) | 0.747 |
| HR < 1 favors migalastat. Adjusted model includes age, sex, and baseline eGFR as covariates.  Abbreviations: CI, confidence interval; ERT, enzyme replacement therapy; FACE, Fabry-associated clinical event; HR, hazard ratio. | | | | |

## Figure S1 | Standardized mean differences and propensity score distributions

Abbreviations: eGFR, estimated glomerular filtration rate; FACE, Fabry-associated clinical event.
